# The Open Arms Healthcare Center’s Integrated HIV Care Services Model

**DOI:** 10.5888/pcd16.180633

**Published:** 2019-10-03

**Authors:** Sandra C. Melvin, June Gipson

**Affiliations:** 1 Open Arms Healthcare Center, Jackson, Mississippi

## Abstract

**Introduction:**

Mississippi has the seventh highest rate of people newly diagnosed with HIV infection, and the city of Jackson — the capital and largest metropolitan area of Mississippi — has the third highest rate of AIDS diagnoses among all metropolitan areas in the nation. Linking patients to care and proper adherence to antiretroviral therapy is important for achieving viral load suppression and reducing transmission of the virus. However, many HIV-infected patients have social and clinical barriers to achieving viral suppression. To overcome these barriers the Open Arms Healthcare Center has implemented an integrated HIV care services model.

**Purpose and Objectives:**

The purpose of this study was to determine whether an integrated model of HIV care influenced linkage to health care, adherence to antiretroviral therapy, and viral load suppression.

**Intervention Approach:**

The integrated HIV care services model consisted of 5 care coordination components: 1) case management, 2) HIV health care (primary health care), 3) behavioral health care (mental and substance abuse screening and treatment), 4) adherence counseling (a pharmacist-led intervention), and 5) social support services (transportation, emergency food assistance, housing, and legal assistance).

**Evaluation Methods:**

We used a cross-sectional research design to examine Open Arms electronic health record data collected from 231 patients from January 2015 through December 2017 to determine if an integrated model of HIV care resulted in increased linkage to health care, higher adherence rates, and improved viral load suppression.

**Results:**

Findings showed a 38.0% increase in the viral load suppression rate, a 12.8% increase in antiretroviral therapy adherence rate, and an 11.0% increase in retention rates among Open Arms patients receiving integrated HIV care.

**Implications for Public Health:**

A comprehensive, holistic approach helps to effectively identify and connect HIV-positive patients to care and relink patients who may have fallen out of care.

**Summary**

**What is already known about this topic?**

Mississippi has the seventh highest rate of people newly diagnosed with HIV infection, and the city of Jackson — the capital and largest metropolitan area of Mississippi — has the third highest rate of AIDS diagnoses among all metropolitan areas in the nation.

**What is added by this report?**

This intervention demonstrates that an integrated model of HIV care involving rapid initiation of treatment combined with wrap-around services results in increased viral load suppression and antiretroviral therapy adherence rates.

**What are the implications for public health practice?**

A comprehensive, evidence-based approach to HIV care that includes access to social support services can result in improved health outcomes for HIV-positive patients.

## Introduction

Data from the Centers for Disease Control and Prevention (CDC) (1) show that HIV diagnoses are not evenly distributed by region in the United States. In 2017, the rate of people who received an HIV diagnosis was highest in the South at 16.1 per 100,000 people, followed by the US 6 dependent areas (American Samoa, Guam, the Northern Mariana Islands, Puerto Rico, the Republic of Palau, and the US Virgin Islands) (12.3/100,000), the Northeast (10.6/100,000), the West (9.4/100,000), and the Midwest (7.4/100,000).

Although the South accounts for only 37% of the US population, 50% of all people living with HIV reside in the South (2). The rate of HIV infection in Mississippi surpasses the rates for 6 of the 50 states. According to the Mississippi State Department of Health (3), black residents make up 37.3% of the state population but account for 72.6% of all Mississippians with HIV (2). Mississippi also has the seventh highest rate of new HIV diagnoses in the nation. Compounding the problem are government policy issues (eg, insufficient program funding), socioeconomic issues (eg, widespread poverty, housing insecurity, lack of access to care), and cultural issues (eg, homophobia, social stigma). Mississippi State Department of Health data (4) show 78% viral suppression among HIV-positive patients, 94% use of antiretroviral therapies, a 65% patient retention rate, and a 17% failure to link into care within the first 6 months of diagnosis.

## Purpose and Objectives

In 2011, researchers (5) observed that for people with HIV infection to fully benefit from antiretroviral therapy, they need to know that they are infected, be engaged in regular HIV care, and receive and adhere to effective antiretroviral therapy. These 3 elements are commonly referred to as the HIV treatment cascade. On the basis of this model, the key measures of the success of any HIV care intervention are linkage to care, retention rate, adherence rate, and viral suppression rate. Therefore, the purpose of our integrated HIV care services model intervention was to evaluate whether a modified integrated model of HIV care would improve linkage to care, retention in care, viral load suppression rates, and antiretroviral therapy adherence rates.

## Intervention Approach

The Open Arms Healthcare Center (Open Arms) is a nonprofit health care organization established in 2013 to provide innovative, holistic, health care services to underserved, underinsured, and underrepresented populations in Mississippi with emphasis on lesbian, gay, bisexual, transgender, and intersex populations. Open Arms links all patients with a preliminary diagnosis of HIV to care on the same day as the HIV diagnosis. Patients are seen by an infectious disease physician and started on antiretroviral therapy during the first visit. The modified HIV care services — the integrated HIV care services model — was implemented by Open Arms in 2015 to provide a holistic, comprehensive approach to HIV care.

The Open Arms program consists of 5 care coordination components: 1) case management, 2) HIV care (primary health care), 3) behavioral health care (mental and substance abuse screening and treatment), 4) adherence counseling (a pharmacist-led intervention), and 5) social support services (transportation, emergency food assistance, housing, and legal services). These components work together to achieve retention in HIV care, antiretroviral therapy adherence, and viral suppression.

**Case management.** The clinical case manager assesses the patient’s medical and psychosocial needs. The completed assessment is designed to give the manager a comprehensive picture of the patient’s complete health care and social support needs. The manager works with patient navigators to coordinate all referrals internal to and external to Open Arms and facilitates all HIV and behavioral health care linkage within 24 hours. Referrals to social support services are made on the same day and are based on the patient’s needs.

**Rapid linkage to HIV care.** Linkage to care is an important first step in successful HIV treatment and is typically defined as the completion of a first medical clinic visit after an HIV diagnosis. It plays a crucial role in the HIV care continuum, because it is a necessary precursor to retention in care, antiretroviral therapy initiation, and viral suppression (6). Therefore, antiretroviral therapy is initiated by the physician at the preliminary diagnosis of HIV infection.

As part of the initial visit, the patient undergoes laboratory testing, which includes a complete blood count, comprehensive metabolic profile, lipid profile, urinalysis, viral load, and genotyping for HIV resistance and testing for CD4, hepatitis (including hepatitis A, B, and C), quantiFERON-TB, G6PD (glucose-6-phosphate dehydrogenase), HLA-B5701 (human leucocyte antigen-B5701), toxoplasmosis, RPR (rapid plasma reagin), and sexually transmitted diseases (syphilis, chlamydia, and gonorrhea). The results are discussed during the initial HIV care visit. Each patient is assigned a case manager who works with the patient to schedule follow-up appointments and identify any potential barriers to care.

**Behavioral health care**. The M3 Checklist is a nationally recognized, peer-reviewed, and clinically validated tool that compiles and evaluates a patient’s potential for mood and anxiety symptoms by using a secure web-based system (7). Each patient completes an initial behavioral assessment by using the M3 checklist. The checklist increases the mental health care provider’s ability to detect and diagnose behavioral health concerns. On the basis of the results of the M3 Checklist assessment, the provider contacts the patient to develop a treatment plan.

**Adherence counseling.** Patients who struggle with adherence are referred to the adherence pharmacist. The pharmacist’s main activities are reviewing laboratory results with physicians and patients, making drug recommendations to physicians, counseling patients on medication side effects and drug–drug interactions, checking pricing of drugs at patients’ preferred pharmacies before new prescriptions are written to ensure that cost is not a barrier to adherence, contacting patients 2 weeks after filling a new prescription to discuss any side effects they may have experienced, and ensuring that patients pick up their prescriptions and are able to incorporate them into their regimen.

**Social support services.** The model provides wrap-around services in the form of internal and external referral services (ie, support groups, transportation, and emergency food assistance; housing, employment services, and mental health services).

## Evaluation Methods

Our summative evaluation used a quasi-experimental, cross-sectional research design to examine Open Arms electronic health record data collected from January 1, 2015, through December 31, 2017, to determine if an integrated model of HIV care resulted in increased linkage to care, increased treatment adherence rates, increased retention rates, and improved viral load suppression. The data were analyzed from September 2018 through October 2018. The study setting was the Open Arms Healthcare Center located in Jackson, Mississippi.

An initial chart review showed that 287 HIV-positive patients used Open Arms services from January 1, 2015, through December 31, 2017. Of these, 231 patients received HIV care at Open Arms and 56 did not. We assessed demographic characteristics of the 231 (Table).

**Table:** Demographic Characteristics, Open Arms Healthcare Center Patients With HIV (N = 231), Jackson, Mississippi, January 2015–December 2017

Characteristic	Percentage		
	2015 (n = 141)	2016 (n = 75)	2017 (n = 74)
**Sex**			
Male	83	91	81
Female	13	9	19
**Age, y**			
17–24	36	40	17
25–44	48	47	67
45–64	16	14	16
≥65	0	0	0
**Race/ethnicity**			
Black	93	88	90
White	3	7	5
Hispanic	4	5	5
**Sexual orientation**			
MSM	76	72	72
Non-MSM	24	28	28

Abbreviations: MSM, men who have sex with men.

### Outcome measures

We used 4 measures to assess effectiveness of the intervention: linkage to care, retention in care, adherence rate, and viral load suppression rate.

**Linkage to care.** Linkage to care measures the percentage of people who received a diagnosis of HIV in a given calendar year who had 1 or more documented viral load tests or CD4+ tests within 30 days (1 month) of diagnosis. Among Open Arms patients, linkage to care was measured by determining the percentage of patients who had at least 1 HIV medical care visit in each 6-month period of the 24-month measurement period and a minimum of 60 days between medical visits.

**Retention in care.** CDC defines retention in care as the percentage of patients diagnosed with HIV who had 2 or more viral load or CD4+ tests, performed at least 3 months apart. As part of the integrated model of care, each patient at Open Arms is scheduled for follow-up appointments every 3 months.

**Adherence rate.** An adherence to antiretroviral therapy of 95% is required as an appropriate level to achieve maximal viral load suppression (8–10) and lower the rate of opportunistic infections (11). The adherence rate was measured as an increase in adherence to antiretroviral therapy among people in HIV medical care.

**Viral load suppression rate.** The viral load suppression rate is defined as the percentage of patients on antiretroviral therapy for a minimum of 12 weeks who had 1 health care visit in each 6-month period of the review period and who were considered suppressed as derived from the last recorded viral load of the review period. Viral load suppression is defined as a viral load of less than 200 copies/mm. CD4+ counts were taken for Open Arms patients every 3 months.

### Data collection and analysis

SPSS version 20.0 (IBM Corp) was used to analyze demographics, linkage to care, antiretroviral therapy adherence rate, retention rates, and viral load suppression rates. Data were collected and stored in Advanced MD, the Open Arms medical record system, and CAREWare (https://hab.hrsa.gov/program-grants-management/careware), a free, electronic health and social support services information system for Ryan White HIV/AIDS Program grant recipients and their providers. CAREWare contains “customizable modules for tracking demographics, services, medications, laboratory test results, immunization history, diagnoses (updated with ICD-10 [International Classification of Diseases, Tenth Revision (12)] codes), referrals to outside agencies, and an appointment scheduler.”

## Results

From January 1, 2015, through December 31, 2017, 231 HIV-positive patients were treated at Open Arms. Because Open Arms uses a rapid initiation model of HIV care, linkage to care for patients was 100%. Patients were immediately assigned to a case manager at the initial diagnosis and scheduled for the first medical visit within 72 hours of their diagnosis. Retention in care increased from 42% in 2015 to 53% in 2017, representing an 11.0% increase over the 3-year period. Falling out of care was defined as missing at least 2 scheduled appointments within one year, relocation, referral to another facility, or incarceration (Figure). The antiretroviral adherence rate for Open Arms patients increased from 82.8% in 2015 to 95.6% in 2017, representing a 12.8% increase over the 3-year period. The viral load suppression rate increased from 59% in 2015 to 81% in 2017, representing a 38% increase over the 3-year period since the model was implemented.

**Figure:**
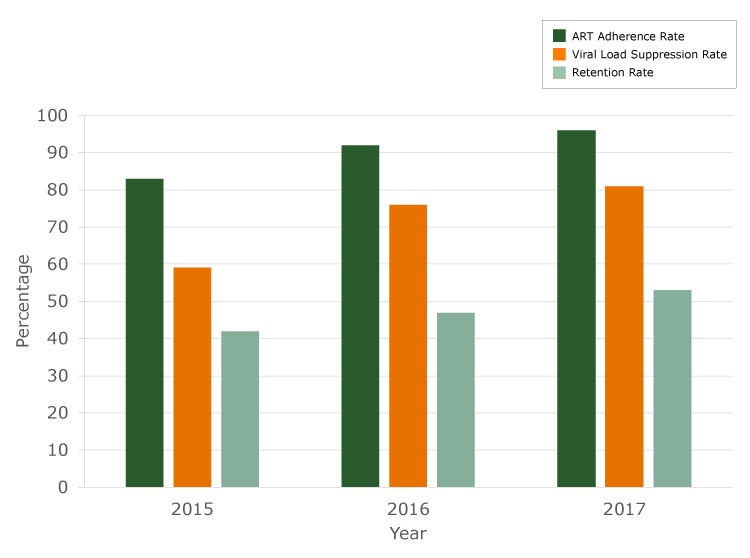
Antiretroviral therapy (ART) adherence rate, viral load suppression rate, and retention rate, Open Arms Healthcare Center integrated HIV care services model, 2015–2017.

**Table:** 

Outcome Measure	2015	2016	2017
Antiretroviral adherence rate, %	83	92	96
Viral load suppression rate, %	59	76	81
Retention rate, %	42	47	53

## Implications for Public Health

Early initiation and linkage to care is necessary to reduce the transmission of HIV and prevent new infections. Without timely entry into care, people with HIV infection miss an opportunity to benefit from HIV treatment at the earliest stage feasible. Linkage to care within 3 months of infection significantly increases the likelihood of achieving viral suppression. Delayed linkage to care is a major barrier to the potential of treatment as a means of prevention to reduce HIV transmission rates in the United States. Therefore, a comprehensive, holistic approach is necessary to effectively identify and connect HIV-positive patients to care and to relink patients who may have fallen out of care. This approach requires consistent follow-up and addressing of social barriers (eg, poverty, transportation, lack of insurance, mental health issues, basic dietary needs) that may impede a patient’s ability to access care consistently. Care and support are important because they facilitate immediate access to treatment when a person is diagnosed with HIV and promote adherence to treatment to attain viral suppression for people living with HIV.

The trend data presented by this study indicate that adherence rates, retention rates, and viral load suppression rates improved when this enhanced model of HIV care was implemented. The data presented in this article are limited in that only information about trends in rates of adherence, retention, and viral load suppression are presented. Future research should evaluate which specific elements of our integrated care model are most associated with viral control and what role provider experience plays in this association. The patient–provider relationship is a very important component of the HIV care continuum. Therefore, understanding the challenges to cultivating and maintaining this relationship is critical for linking and retaining patients in HIV care,

CDC released HIV care guidelines (13) based on the premise that early linkage to care results in improved care as a result of improved antiretroviral therapy adherence. Our intervention demonstrates that rapid initiation of treatment combined with wrap-around services results in increased viral load suppression, increased retention rates, and improved antiretroviral therapy adherence rates. Therefore, a comprehensive, evidence-based approach that includes early linkage to care and wrap-around services is necessary to effectively identify and connect patients to care and to relink patients who may have fallen out of care.
